# A Pilot Study to Develop an Assessment Tool for Dogs Undergoing Trap-Neuter-Release (TNR) in Italy. An Overview on the National Implementation of TNR Programmes

**DOI:** 10.3390/ani11113107

**Published:** 2021-10-30

**Authors:** Greta Veronica Berteselli, Cristina Rapagnà, Romolo Salini, Pietro Badagliacca, Fabio Bellucci, Filomena Iannino, Paolo Dalla Villa

**Affiliations:** 1 Istituto Zooprofilattico Sperimentale dell’Abruzzo e del Molise “G. Caporale”, Via Campo Boario, 64100 Teramo, Italy; Cristina.RAPAGNA@ext.efsa.europa.eu (C.R.); r.salini@izs.it (R.S.); p.badagliacca@izs.it (P.B.); f.bellucci-esterno@sanita.it (F.B.); f.iannino@izs.it (F.I.); p.dallavilla@oie.int (P.D.V.); 2 Department of Veterinary Medicine, University of Milan, Via dell’Università 6, 26900 Lodi, Italy; 3 European Food Safety Authority, Via Carlo Magno 1A, 43126 Parma, Italy; 4 Ministry of Health, Directorate General for Animal Health and Veterinary Medicinal Products, Via G. Ribotta 5, 00144 Roma, Italy; 5 OIE Sub-Regional Representation in Brussels, Boulevard du Jardin Botanique 55, 1000 Bruxelles, Belgium

**Keywords:** animal welfare, dog, inter-observer reliability, test–retest reliability, trap-neutered-release, TNR, welfare assessment

## Abstract

**Simple Summary:**

This paper describes the development of a tool to assess the welfare of dogs recruited in trap-neuter-release (TNR) programmes and the Italian situation involving the implementation of these programmes. The TNR approach has been proposed as an alternative to long-term sheltering to control the rising population of free-roaming dogs. The protocol was developed on the basis of a shelter quality protocol (SQP). The measures included in the protocol were integrated with other welfare indicators proposed in the scientific literature. Nine Italian regions out of 20 (all from central and southern Italy) prescribe by law the implementation of TNR programmes. A varied scenario and some critical issues related to the TNR approach for the management of the dog population emerged. The findings, although preliminary, suggest that the protocol could be a useful tool for the assessment of dog welfare.

**Abstract:**

A descriptive analysis, inter-observer and test–retest reliability of the animal-based measures (ABMs) included in the protocol were performed. This study aimed at the development of a welfare assessment protocol for dogs recruited in the trap-neuter-release (TNR) programmes and the description of the implantation of these programmes in Italy. Nine Italian regions carried out TNR programmes. A varied scenario, along with some critical issues, emerged. Fifty dogs were recruited and assessed simultaneously by two assessors to determine the reliability of ABMs included in the protocol. A subsample of ten dogs were assessed three times to assess test–retest reliability. All females were neutered against 36% of males. Most dogs were adults (58%) and of a large size (68%). Vaccine prophylaxis and parasitic prevention were regular in 13% and 76% of dogs, respectively. Few dogs showed lameness, evidence of pain, other clinical problems, or thermal discomfort. Overall, 82% of dogs did not show fear or aggression to unfamiliar people. The level of agreement between the two assessors was quite high, ranging from substantial (0.61–0.80) to perfect (1) for the majority of measures. This study highlighted some critical issues in TNR implementation and the suitability of the protocol as a tool for animal welfare assessment.

## 1. Introduction

With the enactment of the framework law no. 281 of 14 August 1991 on “Companion animals protection and prevention of stray dogs”, many policy innovations were introduced and the law expressed a new sensitivity towards animals. The law also aimed at solving long-standing issues, including the problem of stray dogs. This law outlined the responsibilities and duties of dog owners and of public institutions and introduced a “no-kill” policy limiting the euthanasia of dogs to those that are “seriously ill, incurable or proven to be dangerous” [1].

This national framework law does not provide standards for the management of stray dogs, which are defined by the twenty regional authorities in Italy (five of which have special statutes). This has generated considerable variability in dog management approaches around the country [2,3,4]. The legislation mandates the following for stray dogs. Those dogs that are caught are placed in quarantine for up to a maximum of 60 days in a shelter. The dogs are microchipped (if not already so identified) and are recorded in the regional registry, treated for any diseases, given appropriate prophylactic treatments (e.g., antiparasitic medications and vaccinations), and are sterilized. After this quarantine period, if they are not returned to their owner or adopted, the dogs are moved to long-term shelters, where they will remain until adoption or until death [4,5].

Some regions in central and southern Italy have also implemented trap-neuter-release programmes (TNR) to control the dog population. At the municipal level, the Local Veterinary Health Unit (LVHU) is responsible for the capture and management of stray dogs (including microchipping, sterilisation and vaccination). All captured dogs must be maintained in the local public shelter for health screenings and treatment. These dogs may be returned to their place of capture under specific conditions: the dog must be sterilised, considered as harmless, and accepted by the community after the designation of an appropriate monitor (a citizen or an animal rights association) who ensures that the dog is cared for. Generally, the dogs undergoing such a programme are legally under the responsibility of the mayor of the municipality where they are released and are not confined to a yard or house. They are able to roam freely. In some regions, such as Lazio, animal rights associations can take over the management and responsibility for these dogs, after obtaining the agreement of the LVHU. TNR is considered an alternative to a long-term shelter, which does not necessarily provide an optimum environment for dogs (confinement combined with novel experiences and changes in routine can be stressful for the sheltered animals) [3,4,6,7,8,9,10,11]. TNR is viewed as a way to lower the costs of maintaining long-term dog shelters that burden administrations. Despite these efforts, the problem of stray dogs is still a challenge in Italy.

Other countries besides Italy, such as Brazil, Bali, Bangladesh, Bahamas, India, Thailand, and some European countries such as Bosnia-Herzegovina, Bulgaria, Greece, Malta, Serbia, Spain, and Ukraine, have implemented TNR programmes to cope with the problem of stray dogs (i.e., the control of rabies transmission) and to stabilize canine populations [12,13,14,15,16,17].

The ubiquitous nature of TNR, its application by leading animal welfare organisations and governments, and the poor welfare implications of alternative dog population control measures all contribute to the perception of TNR as a welfare-friendly method, although welfare assessment is often absent, may rely upon proxy assessment, or be limited to few measures of health status [14,18].

There has been considerable interest in the welfare needs and conditions of dogs maintained in animal shelters, and appropriate shelter protocols have been investigated and recommended [3,4,11,19]. These studies have validated physiological, behavioural and endocrine parameters for welfare assessment [8,20,21,22,23].

The shelter quality protocol (SQP) is one of the tools developed for shelter dogs’ welfare assessment. The SQP took its inspiration from the Welfare Quality^®^ protocol developed for the evaluation of farmed animal welfare. In particular, it emphasized the principles of good nutrition, good housing, good health and appropriate behaviour [3,4,24].

However, to date, only one study aimed to develop a protocol to assess the welfare of dogs submitted to TNR. This report identified the potential welfare issues for dogs during the TNR process and noted that the focus on population control combined with a lack of a standardized approach including animal welfare evaluations may be a risk for individual dogs undergoing TNR [18].

The aims of this pilot study were to develop a new welfare assessment tool for dogs in TNR programmes and to test its inter-observer and test–retest reliability of the animal-based measures (ABMs) included in the protocol. Inter-observer reliability indicates the reproducibility of measurements; specifically, it is the degree to which a measure is free from errors and will therefore yield the same results when repeated. Test–retest reliability tests the stability of the scores of a stable construct obtained from the same person on two or more separate occasions. Reliability assesses the degree to which scores can be distinguished from each other, despite measurement error. In the case of test–retest assessment, intraindividual response variability is used to estimate measurement error [25,26,27]. The data collected were also used to describe how the TNR programmes are implemented in Italy.

## 2. Materials and Methods

### 2.1. Protocol Description

The protocol for the assessment of dogs undergoing TNR was developed using the SQP [3,4,19] as a model. The measures included in the protocol developed for this study included SQP measures, but also other approaches described in the existing scientific literature [11,28,29,30,31]. In particular, affiliative behaviours (i.e., licking the other dog’s muzzle, initiating physical contact, allo-grooming, play bow) and agonistic behaviours (i.e., raised hackles, submissive body posture, teeth baring, biting) were included in the protocol [32,33,34,35]. These behaviours provide information on social interactions and emotions. Agonistic behaviours, such as aggression to conspecifics, have been reported to be associated with stress and poor welfare [6,31,32]. In contrast, affiliative behaviours play an important role in the formation of bonds and alliances among individuals, allowing social interaction, and are essential for maintaining complex social groups [33,34,35]. Special attention was focused on the ABMs, because animal behaviours are considered among the best indicators of animal welfare [36]. The elements of the SQP that were considered include health measures (i.e., BCS—Body Condition Score, skin condition; coat condition; signs of diarrhoea; lameness, coughing, evidence of pain, signs of thermic discomfort, reaction to humans) as well as management-based measures (MBMs) and resource-based measures (RBMs), such as shelter from adverse weather conditions, appropriate bedding materials, feeding regime, and type of food (modified appropriately since the dogs were not housed in shelters). These measures may indicate a risk of welfare problem and provide important information to complement the ABMs [36]. The selection of suitable measures also considered the feasibility of assessment (i.e., practicality under field condition) [3,11,30,37]. Table 1 summarises the different ABMs included in the protocol. 

The assessment protocol contained five parts:

1. General information (i.e., dog identification, information on dog’s responsible person, Global Positioning System (GPS) coordinates);
2. Signalling (i.e., sex, size, age, medical history, social condition);
3. Welfare measures at individual level (ABMs);
4. Available resources and management (MBMs and RBMs) (i.e., presence of shelters, bedding materials, food and water points, feeding regime, presence of garbage nearby); and
5. Living environment (e.g., countryside, settlement) and location features (e.g., garden/field, bushes, parking lot, road/sidewalk).

The protocol was tested under field conditions using a simple and objective scoring system to produce a simple and objective welfare score (WS). The score was represented by a binary 1/0 scoring system including only ABMs. The use of this tool allows the calculation of a WS based on the proportions of existing positive and negative indicators. For example, ‘BCS too thin’, ‘Presence of coughing’, ‘Presence of skin problems’, and ‘Inadequate coat condition’ are negative indicators, whereas ‘BCS adequate’, ‘No signs of fear or aggression’, ‘Presence of affiliative behaviour’, ‘Absence of pain’, and ‘Absence of lameness’ are positive indicators. When a measure cannot be scored, it is marked as not applicable (NA). The NA number is subtracted from the denominator (total number of assessed indicators). The WS can therefore take any value between −1.0 and +1.0. 

A score of −1.0 would occur in a case where none (0%) of the listed positive indicators and only negative indicators are observed. Conversely, a score of +1.0 would occur in a case where all of the scored indicators are positive. A score of 0.0 would indicate that the proportion of positive indicators are the same as the proportion of negative indicators. The dog would be experiencing a neutral welfare condition. When the number of not applicable (NA) measures is greater than six, it is not possible to calculate the WS [11].

### 2.2. Information on Implementation of TNR Programme

**Figure 1:**
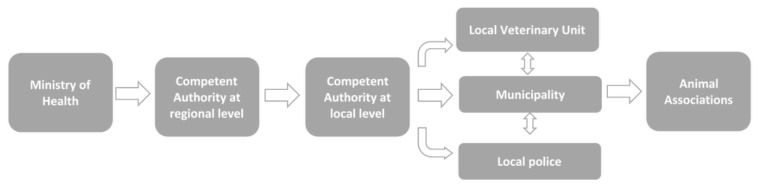
Flow chart to obtain information on TNR programmes implemented in Italy and on dogs involved in these programmes.

The Italian regions that implement a TNR programme as an alternative to long-term shelters were identified via an analysis of the regional laws regarding companion animal protection and the prevention of stray dogs. Regional authorities, municipalities, local authorities, and animals’ rights associations were contacted to obtain information about the status of TNR programmes and the number of dogs involved. In addition, these organizations and institutions were asked for details about the geo-localization of the dogs, and other contacts required organising on-site visits for welfare assessment. In Figure 1, the process to obtain this kind of information is outlined.

### 2.3. Statistical Analyses

A descriptive analysis of the collected data was undertaken to document the implementation of TNR programmes in Italy as well as the welfare condition of dogs. The prevalence of ABMs, RBMs and MBMs were calculated.

An inter-observer agreement was analysed to assess the reliability of the ABMs included in the protocol. Inter-observer reliability was evaluated using Cohen’s kappa for qualitative variables; these variables were all categorical [38].

The fifty free-roaming dogs surveyed in this study were from Abruzzi, Basilicata and Apulia. The dogs were recruited through animal advocacy organizations, and the visits were organized according to availability of the members of organizations or of the person in charge of the dog. Dogs were assessed in the field by two different assessors simultaneously and independently. Generally, the person responsible for the dog or belonging to the association accompanied the assessors in the living environment and identified the dogs subjected to TNR. When it was possible, the recognition was performed through microchip reading. The two assessors were both women, aged between 28 and 42 years, with specific expertise in animal welfare. Both assessors were experienced dog observers and had used the SQP. They were therefore familiar with the welfare assessment protocol approach. An evaluation began when the assessor stood next to (at a maximum of 2 m) the dog subject but without interacting with it (unless required by the protocol). When necessary, the assessors followed the dogs if they moved, performing the assessment according to the described procedure. All the assessments were carried out during daylight hours.

Among the ABMs, a short behavioural test was carried out with the aim of assessing the dogs’ reactions to unfamiliar people. The assessment used was a partially modified SQP which was subdivided into two stages, permitting the reactions of the dogs to be recorded. In this pilot study, the assessor approached all the selected dogs (one at the time), ignored them for 30 s, and then crouched down, talking gently to the dog for 30 s. The assessment of RBMs and MBMs were performed after the assessment of ABMs. The whole assessment was carried out independently by the two assessors.

To explore the test–retest reliability of measures, the same person assessed ten dogs three times with a two and four-week interval among scoring sessions, respectively, following the procedure described previously. Fleiss K analysis was performed [39]. The level of significance was set at 0.002 after applying the Bonferroni correction. For all analyses, z scores and *p* values were also computed to determine whether agreement was greater than could be expected by chance alone. According to Landis and Kock (1977), the agreement levels for Kappa values (k) are as follows:

- 0.00, less than chance agreement;
- 0.01–0.20, slight agreement;
- 0.21–0.40, fair agreement;
- 0.41–0.60, moderate agreement;
- 0.61–0.80, substantial agreement;
- 0.81–0.99, almost perfect agreement; and
- 1, perfect agreement [40].
- Statistical analyses were carried out using R V.2.15.3 [41].

## 3. Results

### 3.1. TNR Programme Implementation in Italy

Nine Italian regions out of 20 (all from central and southern Italy: Abruzzi, Basilicata, Campania, Calabria, Lazio, Molise, Apulia, Sardinia and Sicily) prescribe by law the implementation of a TNR programme (Table 2). Generally, gathering information on the implementation of these programmes was very challenging.

In some cases, information could not be collected due to the difficulty of connecting with the appropriate authorities. In some cases, it was impossible to identify the competent authority who possessed the requested information, even though, by law, the dogs in a TNR programme must have their health and welfare monitored by qualified personnel identified by the municipality. Often, animal advocacy organizations were the only ones able to give useful information on the dogs submitted to TNR because they were directly involved in the field management of the dogs. When the access to regional canine registries was allowed, the data were often outdated or incomplete, and this made it impossible to obtain reliable information about the number of dogs submitted to the TNR programmes or to identify how they were managed or where they were located. Moreover, in some cases, it was not possible to differentiate the stray dogs submitted to TNR from privately owned dogs because they were registered under the name of the private citizen who was the monitor, without any other information or labelling. In addition, dog deaths were not always registered, so the number of dogs in the registry could be an overestimate.

As a result of this investigation, a diversified and varied scenario emerged. Each of the nine regions legislating for TNR had a different approach to the TNR programme’s implementation that could also change from municipality to municipality. The terminology for these dogs varies and includes “community dogs”, “local dogs”, “dog returned to territory”, and “dog released on the territory”. The differences in terminology seem to be based on whether or not a monitor is identified. For convenience, the expression “free-roaming dogs in the territory” (FRDT) will be used to indicate all the dogs that have undergone Italian TNR programmes. This definition does not include free-roaming unowned dogs (e.g., feral dogs) and free-roaming owned dogs (e.g., pet dog free to roam uncontrolled). Generally, these dogs are not in compliance with local regulatory requirements (not officially identified by microchip and not registered in the regional canine registries) [42].

### 3.2. Welfare Assessment: Descriptive Analysis

The recruitment of FRDT for this project was made more difficult by the challenge of locating appropriate animals that had undergone TNR in parallel with the poor collaboration of competent regional authorities.

Fifty dogs, from the Abruzzi, Basilicata and Apulia regions, half males and half females, were recruited for the project and assessed by the protocol. The females were all neutered, but only 36% (9/25) of males were castrated; the remaining males had only undergone a clinical examination and vaccination before release. Most of the FRDT (29 or 58%) were estimated to be between 3 and 7 years old. Only four (8%) were young (7–24 months) and the remaining 34% were older than 7 years. Thirty-four (68%) of the FRDT were large (>21 kg), 26% were medium (11–20 kg) and 6% were small (dog size has been estimated by approximation). The prevalence of FRDT living in pairs or in a group was 66%, whereas 34% were alone.

Only 13% of FRDT were vaccinated regularly; most of them were vaccinated only when caught (69%), and 16% of dogs had never been subjected to prophylactic vaccination. For 2% of dogs this information was not available. However, anti-parasite treatments were carried out regularly on most subjects (76%). Of the remaining FRDT, 12% had never been subjected to anti-parasite treatment or had only received it irregularly (12%). Generally, treatment was left to animal advocacy organizations. In Table 3, the results of ABMs were summarised.

In terms of resources for the dogs, shelter from the weather was available for 76% of FRDT assessed. Bedding materials such as kennels, baskets, blankets or platforms were available for 74% of FRDT. Refreshment points were present in most cases (74%) at the moment of assessment. The hygienic conditions of the food/water bowls were adequate in 52% of cases. Garbage was observed nearby in 2% of cases. The majority of FRDT (52%) were fed with a mixed diet composed of commercial food (pellets and/or cans) plus cooked food and/or food waste. For 46% of FRDT, the diet only included commercial food. Overall, 2% of FRDT are fed only with cooked food. Feeding is guaranteed daily for all dogs at least once a day. Often, people living in the same neighbourhood bring food for the FRDT. As regards the location of the FRDT, 48% were observed in the rural areas outside human settlements, 36% were living in settlements and the remaining 16% were observed in the parking areas around shopping malls or in the industrial zone. Most FRTD (58%) were observed on a street or sidewalk, 14% were inside the courtyard of a building and 10% were in a field. The remainder were observed in the parking lots (8%), in the bushes (6%), or in the town square (4%).

### 3.3. Welfare Assessment: Reliability of Measures

#### 3.3.1. Inter-Observer Reliability

After analysing the qualitative variables, the Cohen’s Kappa analysis showed a good level of agreement between the two observers, ranging from substantial (0.61–0.80) to perfect (1) for the majority of variables. A perfect agreement (k = 1) was obtained for the following measures: “Body condition”, “Affiliative behaviours”, “Signs of diarrhoea” and “Evidence of pain”. The measures for “Other clinical problems”, “Coat condition”, “Reaction to unfamiliar people”, and “Lameness” produced an almost perfect agreement. “Thermal discomfort” (k = 0.66) showed substantial agreement. It was not possible to calculate the correlation for “Agonistic behaviours” and “Coughing” because of the lack of variability in the data. All *p* levels were significant (*p* < 0.001), resulting in a rejection of the hypothesis that the agreement reached was simply due to chance (Table 4).

#### 3.3.2. Test–Retest Reliability

For measures such as “Agonistic behaviours”, “Skin problems”, “Signs of diarrhoea”, “Thermal discomfort”, “Evidence of pain”, “Coughing” and “Lameness”, the statistical analysis could not be carried out due to the high homogeneity of the sample. Agreement was not achieved for the measures “Other clinical problems” and “Affiliative behaviours” (*p* levels were not significant). For “BCS” and “Coat condition”, the observations were in perfect agreement (k = 1). Assessment of “Reaction to unfamiliar people” resulted in substantial agreement (k = 0.65). For these measures, the *p* levels were significant (*p* < 0.001), leading to the rejection of the hypothesis that the agreement reached was simply due to chance.

### 3.4. Welfare Assessment: Welfare Score

The average WS of the sample of dogs analysed was 0.69 (ranging from 0.2 to 1), showing a level of dog welfare in the upper quadrant. No FRDT returned a negative WS, while only three subjects (6%) scored a perfect 1. The majority of FRDT (42%) received a WS greater than 0.8 but less than 1, whereas 28% of FRDT were scored as greater than 0.5 but less than 0.8. The remaining 20% of FRDT received a WS between 0.2 and 0.5. 

## 4. Discussion

An alternative approach, previewed by Italian law 281/1991, to housing the dogs in shelters is the “conversion” of stray dogs to “community dogs” by TNR [1,2,14]. TNR programmes are considered by some competent authorities as being an appropriate tool to control and manage roaming dogs, dog zoonoses and human–animal conflicts [31,42]. Despite the TNR approach being considered a welfare-friendly method, it is not free from risks for the dogs subjected to these programmes, and a welfare assessment is often absent [31].

The protocol developed in this pilot study was designed to assess the welfare of FDRT that have undergone TNR programmes. The protocol takes inspiration from the SQP, a tool validated for the welfare assessment of dogs in shelters [3]. The current protocol for the welfare assessment of FRDT takes advantage of the ease of assessment of animal-based measures (ABMs—both health and behavioural measures) that are integrated with RBMs and MBMs. A preference was given to ABMs because they permit the real-time assessment of animal welfare [36].

Moreover, the proposed scoring system resulted in a single, objective measure of the welfare condition of the animal [11,43]. 

The consistent level of agreement between the two assessors, who evaluated a sample of fifty FRDT, showed the reliability of the ABMs included in the assessment protocol specifically created and designed for FRDT welfare. The protocol can provide a possible way to assess the welfare of dogs subjected to TNR. This approach could be applied for the assessment of stray dogs’ welfare as well. Further studies need to confirm the results.

On the basis of the results, some considerations can be made. Some limitations emerged during in-field activities. The evaluation of behavioural measures could be affected by the environmental condition during the assessment. The dogs were not confined during the assessment, and they could move, run away or hide. Moreover, if the behaviours occur very quickly, it could be difficult to record each occurrence reliably. Similarly, if the assessment timing is too short, rare or specific behaviours such as agonistic or affiliative behaviours may not be observed. Finally, the reliability of the recording may be influenced by the clarity of the definition of the behavioural category or measurements [11,19]. 

Although the dog sample recruited for this study is not representative of Italian FRDT, none of the fifty dogs assessed in the study were scored as having poor welfare. This is a significant finding. The presence of shelters for the dogs (to escape adverse weather conditions) provided with bedding and close to feeding stations indicates that appropriate environmental conditions are made available to all the dogs. The dog’s primary needs are guaranteed. Access to food and water may explain why, in most cases, dogs were not observed near garbage resources [29]. Moreover, most dogs did not show aggressive or fear-related behaviour towards the assessors. In addition to the common problems associated with the presence of free-roaming dogs and cats in a community, one must also add public health and welfare issues [14,42,44,45,46,47,48,49]. Indeed, if their needs are not guaranteed and health surveillance and prophylaxis are not implemented correctly, the welfare of these dogs can be threatened [13,16,50,51]. Appropriate awareness campaigns and education of local citizens are important elements leading to the success of TNR programmes [15,17].

In the present study, the dog population was composed primarily of adult (58%) and aging dogs (34%), and dogs of a large size (68%). The dogs were evenly divided by gender. General sufficient welfare level guaranteed by the management of these dogs may promote better quality of life and consequently an increase in life expectancy. However, it is important to consider that an elderly age, male gender, along with large size are described as risk factors for abandonment [52].

In Italy, the TNR programmes are currently subject to different regional regulations. Collecting adequate information on the TNR programme and identifying those responsible for health checks and dogs care has proved to be very difficult. The survey on the distribution and measurement consistency of Italian FRDT did not produce reliable data. The regional canine registries are not consistently updated for dog deaths. Dogs are automatically deleted from the registries at the end of the twentieth year of registration. 

The fragmentation of information did not permit a clear description of how TNR programmes are implemented in Italy, nor did it permit an assessment of the welfare of dogs that have undergone TNR. However, in our opinion, the difficulties encountered in collecting data on FRDT have highlighted problems in the management of TNR.

A comprehensive dog population management programme must include control of unowned and owned roaming dogs. A primary task must be the estimation of the number, distribution and ecology of owned and unowned dogs and the identification of the sources of stray dogs (i.e., abandoned dogs, owned dogs free to roam, unowned dogs that reproduce successfully) [42,44]. Raising public awareness regarding the need for the registration and identification of dogs, for fertility control and for veterinary care should also be addressed [15,43,49]. The accuracy of the dog registry is uncertain, because there is no active control on dog identification and registration. The role of the dog registry in epidemiological and dog population control is still underestimated [43]. Monitoring dog populations is considered an ethical, ecological, and economic requirement to control and eradicate disease outbreaks, to improve animal welfare and to control the canine population. Other approaches—such as euthanasia or culling—are ethically unacceptable and are prohibited by law in Italy [42].

TNR is one approach for dog population management, but it must be used in combination with other strategies to form a well-functioning dog control system. An effective approach to managing dog populations is to have an adequate proportion of the dogs in a given population sterilized [49]. As demonstrated by the result of this pilot study, most male dogs released into the Italian territory are not castrated (64%). By the contrast, females are all spayed. The substantial difference found in the percentage of sterilised dogs related to their gender should be better explored. Although the sterilization of dogs is mandatory in Italian TNR programmes, available resources (funds and personnel) or cultural and social issues could influence the male sterilization rate, as found in studies on pet owners [53,54]. Further studies are needed to deepen this topic.

Population simulation models estimate that the effect of neutering female dogs is much more significant than the effect of castrating males, in terms of population size reduction [55]. The percentage of females that needs to be sterilised per year depends on the potential population growth rate (the number of dogs that will be in the population after one year, compared to the original number of dogs) [49]. A 70 percent sterilization rate is necessary to stabilize dog populations. Until the proportion of breeding females is less than 20 percent, dog overpopulation will continue to be a problem [16].

The practice of sterilization is widely considered a good one in dog population management, and it is often carried out through the most appropriate TNR programmes, although recent studies have questioned the risks and benefits of this practice in relation to the welfare of individual animals [56]. A beneficial effect of sterilization on lifespan is more consistently demonstrated in females (i.e., a reduction in mammary cancer) than in males [57,58]. Recent studies in pets show that several pathologies (i.e., prostatic carcinoma, urinary incontinence in females, musculoskeletal disease, behavioural problems) can be potential long-term effects of surgical sterilization [56,57,59,60,61].

TNR programmes are focused on the control of a whole canine population principally, which must meet targets in terms of neutering dogs, rather than individual dog welfare. Individual dog welfare may, therefore, be disregarded, and this may be detrimental for individual dogs [18,31]. 

To our knowledge, this is the first study on data collected on Italian TNR programmes at the national level to assess and monitor the welfare of dogs involved in those programmes by the application of a protocol specifically developed for this purpose. Most studies were principally conducted at the regional level, and the dog welfare assessment was often absent or based on health indicators only [15,17,44]. Since animal welfare is the outcome of multifactorial effects, behavioural measures in association with health and physiological measures allow a robust animal welfare assessment [3,4,11,19,31,43].

## 5. Conclusions

The welfare of dogs undergoing TNR should be considered an important parameter. This paper describes a new welfare assessment tool for the assessment of dogs undergoing TNR. The protocol is based on the SQP. It includes measures involving animals, resources and management. The animal-based measures were considered the best approach to assess animal welfare and were based on studies in the recent literature. A welfare score resulting from the application of the protocol permitted delivered objective information on the welfare status of FRDT. Further investigations (i.e., increasing the number of samples and observers) and the inclusion of public health measures (i.e., zoonoses, biting events, dog and cat nuisance complaints and environmental contamination) are needed in order to confirm the present results, to refine the protocol’s measures and to mitigate the limitation of binary codes of measures. The data collected through this protocol can be also used to identify hazards related to the management of these dogs and to develop standardised guidelines for TNR programme according to animal welfare. It would be useful to compare the welfare scores for FRDT with stray dogs that have not undergone TNR, as this tool may also be suitable for the assessment of these subjects.

This study was unable to describe in any detail what is happening as regards TNR and dog management in the various regions of Italy. Although TNR is recognised as an important approach in dog population management and is widely implemented by many countries (e.g., Brazil, Greece, Ukraine, Bulgaria, Spain, etc.), the promotion of responsible dog ownership among the population through awareness campaigns and school education programmes, a greater involvement of private veterinarians as a co-operative part of the system, and establishing an effective monitoring system by competent authorities should be additional steps in order to reduce and control the canine population in Italy.

Moreover, the Italian law should be reformed to include a clear definition of the roles of local governments and veterinary health services in managing stray dogs and FRDT. The revised law should mandate the reporting of systematic and reliable data on the actual size of the FRDT population and its distribution so that it would be possible to monitor TNR implementation and the welfare of animals. 

## Figures and Tables

**Table 1:** Animal-based measures (ABMs) included in the protocol. CatV, categorical variable. Y-N, yes, no. When the assessment of a measure was not possible it was scored “not applicable” (NA).

Qualitative Variables	Definition	Type	Score
Affiliative and/or playing behaviours	Any form of intraspecific positive behaviours (e.g., allo-grooming, touching, play bow)	CatV	Y-N
Agonistic behaviours	Any form of intraspecific behaviour relating to aggression or fear (e.g., raised hackles, submissive body posture, teeth baring, growling, biting, physical aggression)	CatV	Y-N
Body Condition	Nutritional status of dog (body condition score)	CatV	Adequate Too thin Too fat
Coat condition	Shiny/clean coat or dull/dirty/ruffed coat	CatV	Adequate Inadequate
Evidence of pain	Protecting or recumbent position, apathy, lethargy, non-reactive to stimuli	CatV	Y-N
Lameness	Evidence of lameness due to foot wounds or other painful disease or amputation.	CatV	Y-N
Other clinical problem	Evidence of nasal/ocular/vaginal discharge, oral/ear lesion, neurologic symptoms, or any other clinical problems.	CatV	Y-N
Reaction to unfamiliar people	Aggressive, fear or neutral/social reaction vs. unfamiliar people (assessors)	CatV	Sociable Only fear Offensive/defensive aggression
Signs of diarrhoea	Direct observation of liquid manner emission or soiling of the perineum	CatV	Y-N
Signs of thermal discomfort	Evidence of polypnea, trembling, shivering huddling	CatV	Y-N
Skin problems	Evidence of wounds, hair loss areas, inflammation areas, dermatitis, ectoparasites, swellings	CatV	Y-N

**Table 2:** Different approaches of the Italian regions to dog population control (DPC).

Region	Macroregion	Statute	DPC Approach	Region	Macroregion	Statute	DPC Approach
Abruzzi	Centre	Ordinary	Shelter + TNR programme	Molise	South	Ordinary	Shelter + TNR programme
Basilicata	South	Ordinary	Shelter + TNR programme	Piedmont	North-West	Ordinary	Shelter
Calabria	South	Ordinary	Shelter + TNR programme	Apulia	South	Ordinary	Shelter + TNR programme
Campania	South	Ordinary	Shelter + TNR programme	Sardinia	Island	Autonomous	Shelter + TNR programme
Emilia-Romagna	North-East	Ordinary	Shelter	Sicily	Island	Autonomous	Shelter + TNR programme
Friuli-Venezia Giulia	North-East	Autonomous	Shelter	Trentino-Alto Adige	North-East	Autonomous	Shelter
Lazio	Centre	Ordinary	Shelter + TNR programme	Tuscany	Centre	Ordinary	Shelter
Liguria	North-West	Ordinary	Shelter	Umbria	Centre	Ordinary	Shelter
Lombardy	North-West	Ordinary	Shelter	Valle d’Aosta	North-West	Autonomous	Shelter
Marche	Centre	Ordinary	Shelter	Veneto	North-East	Ordinary	Shelter

**Table 3:** Results of Animal-based measures assessment. The assessment follows the listed order. NA not applicable.

ABMs Animal-Based Measures	
BCS	
Adequate	68%
Too heavy	4%
Too thin	24%
NA	4%
Coat condition	
Adequate	81%
Inadequate	17%
NA	2%
Skin problems	
Absent	70%
Present	20%
NA	10%
Signs of other clinical problems	
Absent	82%
Present	12%
NA	6%
Lameness	
Absent	73%
Present	11%
NA	16%
Signs of diarrhoea	
Absent	81%
Present	0%
NA	19%
Evidence of pain	
Absent	96%
Present	2%
NA	2%
Thermal discomfort	
Absent	94%
Present	4%
NA	2%
Coughing	
Absent	98%
Present	0%
NA	2%
Reaction to unfamiliar people	
No signs of fear or aggression (sociable/neutral)	82%
Only fear	14%
Aggressive (offensive and defensive aggression)	4%
Affiliative behaviours	
Absent	58%
Present	8%
NA	34%
Agonistic behaviours	
Absent	66%
Present	0%
NA	34%

**Table 4:** Interobserver agreement.

Qualitative Variable	Cohen’s Kappa Value
Other clinical problems	0.91 ^a^
Body Condition Score	1 ^a^
Affiliative behaviours	1 ^a^
Coat conditions	0.93 ^a^
Skin problems	0.78 ^a^
Fear/aggression test to unfamiliar people	0.95 ^a^
Signs of diarrhoea	1 ^a^
Signs of thermal discomfort	0.66 ^a^
Evidence of pain	1 ^a^
Lameness	0.89 ^a^

^a^ z-score. *p* < 0.001. Level of agreement according to Landis and Kock (1977): 0.00, less than chance agreement; 0.01–0.20, slight agreement; 0.21–0.40, fair agreement; 0.41–0.60, moderate agreement; 0.61–0.80, substantial agreement; 0.81–0.99, almost perfect agreement; 1, perfect agreement.

## Data Availability

The data presented in this study are available on request from the corresponding author.

## References

[1] Italian Law (1991). Legge n.281 of 4 Agosto 1991. In Materia di Animali D’affezione e Prevenzione al Randagismo. Gazzetta Ufficiale, n. 203, 30 Agosto 1999. https://www.gazzettaufficiale.it/atto/serie_generale/caricaDettaglioAtto/originario?atto.dataPubblicazioneGazzetta=1991-08-30&atto.codiceRedazionale=091G0324&elenco30giorni=false.

[2] Volsarova E., Passantino A. (2012). Stray dog and cat laws and enforcement in Czech republic and in Italy. Ann. Dell’Ist. Super. Sanitã.

[3] Barnard S., Pedernera C., Candeloro L., Ferri N., Velarde A., Dalla Villa P. (2016). Development of a new welfare assessment protocol for practical application in long-term dog shelters. Vet. Rec..

[4] Arena L., Berteselli G.V., Lombardo F., Candeloro L., Dalla Villa P., De Massis F. (2019). Application of a welfare assessment tool (Shelter Quality Protocol) in 64 Italian long-term dogs’ shelters: Welfare hazard analysis. Anim. Welf..

[5] Barnard S., Chincarini M., Di Tommaso L., Di Giulio F., Messori S., Ferri N. (2015). Free-roaming dogs control activities in one Italian province (2000–2013): Is the implemented approach effective?. Maced. Vet. Rev..

[6] Beerda B., Schilder M.B., Van Hooff J.A., De Vries H.W., Mol J.A. (2000). Behavioural and hormonal indicators of enduring environmental stress in dogs. Anim. Welf..

[7] Hennessy M.B., Voith V.L., Mazzei S.J., Buttram J., Miller D.D., Linden F. (2001). Behavior and cortisol levels of dogs in a public animal shelter, and an exploration of the ability of these measures to predict problem behavior after adoption. Appl. Anim. Behav. Sci..

[8] Hiby E.F., Rooney N.J., Bradshaw J.W. (2006). Behavioural and physiological responses of dogs entering re-homing kennels. Physiol. Behav..

[9] Titulaer M., Blackwell E.J., Mendl M., Casey R.A. (2013). Cross sectional study comparing behavioural, cognitive and physiological indicators of welfare between short and long term kennelled domestic dogs. Appl. Anim. Behav. Sci..

[10] Taylor K.D., Mills D.S. (2007). The effect of the kennel environment on canine welfare: A critical review of experimental studies. Anim. Welf..

[11] Kiddie J.L., Collins L.M. (2014). Development and validation of a quality of life assessment tool for use in kennelled dogs (Canis familiaris). Appl. Anim. Behav Sci..

[12] Tasker L. (2007). Stray Animal Control Practices (Europe).

[13] Smith L.M., Hartmann S., Munteanu A.M., Dalla Villa P., Quinnell R.J., Collins L.M. (2019). The Effectiveness of Dog Population Management: A Systematic Review. Animals.

[14] Dalla Villa P., Kahn S., Stuardo L., Iannetti L., Di Nardo A., Serpell J.A. (2010). Free-roaming dog control among OIE-member countries. Prev. Vet. Med..

[15] Høgåsen H.R., Er C., Di Nardo A., Dalla Villa P. (2013). Free-roaming dog populations: A cost-benefit model for different management options, applied to Abruzzo, Italy. Prev. Vet. Med..

[16] Jackman J., Rowan A., Salem D.J., Rowan A.N. (2007). Free-roaming dogs in developing countries: The benefits of capture, neuter, and return programs. The State of the Animals.

[17] Paolini A., Romagnoli S., Nardoia M., Conte A., Salini R., Podaliri Vulpiani M., Dalla Villa P. (2020). Study on the Public Perception of “Community-Owned Dogs” in the Abruzzo Region, Central Italy. Animals.

[18] Bacon H., Walters H., Vancia V., Connelly L., Waran N. (2019). Development of a Robust Canine Welfare Assessment Protocol for Use in Dog (Canis Familiaris) Catch-Neuter-Return (CNR) Programmes. Animals.

[19] Berteselli G.V., Arena L., Candeloro L., Dalla Villa P., De Massis F. (2019). Interobserver agreement and sensitivity to climatic conditions in sheltered dogs’ welfare evaluation performed with welfare assessment protocol (Shelter Quality protocol). J. Vet. Behav..

[20] Dalla Villa P., Barnard S., Di Fede E., Podaliri M., Candeloro L., Di Nardo A., Podaliri Vulpiani M., Siracura C., Serpell J.A. (2013). Behavioural and physiological responses of shelter dogs to long-term confinement. Vet. Ital..

[21] Cafazzo S., Maragliano L., Bonanni R., Scholl F., Guarducci M., Scarcella R., Di Paolo M., Pontier D., Lai O., Carlevaro F. (2014). Behavioural and physiological indicators of shelter dogs’ welfare: Reflections on the no-kill policy on free-ranging dogs in Italy revisited on the basis of 15 years of implementation. Physiol. Behav..

[22] Part C.E., Kiddie J.L., Hayes W.A., Mills D.S., Neville R.F., Morton D.B., Collins L.M. (2014). Physiological, physical and behavioural changes in dogs (Canis familiaris) when kennelled: Testing the validity of stress parameters. Physiol. Behav..

[23] Righi C., Menchetti L., Orlandi R., Moscati L., Mancini S., Diverio S. (2019). Welfare Assessment in Shelter Dogs by Using Physiological and Immunological Parameters. Animals.

[24] Welfare Quality® (2009). Welfare Quality® Assessment Protocol for Cattle.

[25] Hays R.D., Anderson R., Revicki D. (1993). Psychometric considerations in evaluating health-related quality of life measures. Qual. Life Res..

[26] Taylor K.D., Mills D.S. (2006). The development and assessment of temperament tests for adult companion dogs. J. Vet. Behav..

[27] Martin P., Bateson P. (1993). Measuring Behaviour, an Introductory Guide.

[28] Bonanni R., Cafazzo S., Valsecchi P., Natoli E. (2010). Effect of affiliative and agonistic relationships on leadership behaviour in free-ranging dogs. Anim. Behav..

[29] Barnard S., Ippoliti C., Di Flaviano D., De Ruvo A., Messori S., Giovannini A., Dalla Villa P. (2015). Smartphone and GPS technology for free-roaming dog population surveillance-a methodological study. Vet. Ital..

[30] Pal S.K. (2015). Factors influencing intergroup agonistic behaviour in free-ranging domestic dogs (Canis familiaris). Acta Ethol..

[31] Bacon H., Vancia V., Walters H., Waran N. (2017). Canine trap-neuter-return: A critical review of potential welfare issues. Anim. Welf..

[32] Beerda B., Schilder M.B., Van Hooff J.A., De Vries H.W., Mol J.A. (1999). Chronic stress in dogs subjected to social and spatial restriction. I. Behavioral responses. Physiol. Behav..

[33] Boissy A., Manteuffel G., Jensen M.B., Moe R.O., Spruijt B., Keeling L.J., Winckler C., Forkman B., Dimitrov I., Langbein J. (2007). Assessment of positive emotions in animals to improve their welfare. Physiol. Behav..

[34] Bauer E., Ward C., Smuts B. (2009). Play like a puppy, play like a dog. J. Vet. Behav..

[35] del Toro C.J., Nekaris K.I., Vonk J., Shackelford T. (2019). Affiliative Behaviors. Encyclopedia of Animal Cognition and Behavior.

[36] (2012). EFSA Panel on Animal Health and Welfare (AHAW) Statement on the use of animal-based measures to assess the welfare of animals. EFSA J..

[37] Williams D.L., Hogg S. (2016). The health and welfare of dogs belonging to homeless people. Pet Behav. Sci..

[38] Cohen J. (1968). Weighted kappa: Nominal scale agreement with provision for scaled disagreement or partial credit. Psychol. Bull..

[39] Fleiss J. (1971). Measuring nominal scale agreement among many raters. Psychol. Bull..

[40] Landis J.R., Koch G.G. (1977). The measurement of observer agreement for categorical data. Biometrics.

[41] R Core Team (2020). R: A Language and Environment for Statistical Computing.

[42] World Organisation for Animal Health (2019). Stray Dog Solution Control. Terrestrial Animal Health Code.

[43] Spoolder H., De Rosa G., Horning B., Waiblinger S., Wemelsfelder F. (2003). Integrating parameters to assess on-farm welfare. Anim. Welf..

[44] Carvelli A., Scaramozzino P., Iacoponi F., Condoleo R., Della Marta U. (2020). Size, demography, ownership profiles, and identification rate of the owned dog population in central Italy. PLoS ONE.

[45] Matter H., Daniels T., Macpherson C.N.L., Meslin F.X., Wandeler A.I. (2000). Dog ecology and population biology. Dogs, Zoonoses and Public Health.

[46] Slater M.R. (2002). Community Approaches to Feral Cats: Problems, Alternatives, and Recommendations.

[47] Slater M.R., Rochlitz I. (2005). The welfare of feral cats. The Welfare of Cats.

[48] Cortes S., Alfonso M.O., Alves-Pires C., Campino L. (2007). Stray dogs and leishmaniasis in urban areas, Portugal. Emerg. Infect. Dis..

[49] International Companion Animal Management Coalition (ICAM) Coalition (2008). Guideline Document. Humane Dog Population Management Guidance. https://www.icam-coalition.org/wp-content/uploads/2019/09/2019-ICAM-DPM-guidance-Interactive-updated-15-Oct-2019.pdf.

[50] Belsare A.V., Gompper M.E. (2013). Assessing demographic and epidemiologic parameters of rural dog populations in India during mass vaccination campaigns. Prev. Vet. Med..

[51] Tenzin T., Ahmed R., Debnath N.C., Ahmed G., Yamage M. (2015). Free-Roaming Dog Population Estimation and Status of the Dog Population Management and Rabies Control Program in Dhaka City, Bangladesh. PLoS Negl. Trop. Dis..

[52] Fatjó J., Bowen J., García E., Calvo P., Rueda S., Amblás S., Lalanza J.F. (2015). Epidemiology of dog and cat abandonment in Spain (2008–2013). Animals.

[53] Faver C.A. (2009). Sterilization of companion animals: Exploring the attitudes and behaviors of Latino students in South Texas. J. Appl. Anim. Welf. Sci..

[54] Downes M.J., Devitt C., Downes M.T., More S.J. (2015). Neutering of cats and dogs in Ireland; pet owner self-reported perceptions of enabling and disabling factors in the decision to neuter. PeerJ.

[55] Dugassa J., Fromsa A., Wirtu A. (2020). The role of canine surgical sterilization and other dog population management strategies to complement rabies prevention and control programs. JAH.

[56] Bryan J.N., Keeler M.R., Henry C.J., Bryan M.E., Hahn A.W., Caldwell C.W. (2007). A population study of neutering status as a risk factor for canine prostate cancer. Prostate.

[57] McKenzie B. (2010). Evaluating the benefits and risks of neutering dogs and cats. CAB Rev..

[58] Garde E., Pérez G.E., Vanderstichel R., Dalla Villa P.F., Serpell J.A. (2016). Effects of surgical and chemical sterilization on the behavior of free-roaming male dogs in Puerto Natales, Chile. Prev. Vet. Med..

[59] Sparkes J., Körtner G., Ballard G., Fleming P.J.S., Brown W.Y. (2014). Effects of Sex and Reproductive State on Interactions between Free-Roaming Domestic Dogs. PLoS ONE.

[60] McGreevy P.D., Wilson B., Starling M.J., Serpell J.A. (2018). Behavioural risks in male dogs with minimal lifetime exposure to gonadal hormones may complicate population-control benefits of desexing. PLoS ONE.

[61] Starling M., Fawcett A., Wilson B., Serpell J., McGreevy P. (2019). Behavioural risks in female dogs with minimal lifetime exposure to gonadal hormones. PLoS ONE.

